# Mesenchymal stromal cells 2.0: thinking outside the box

**DOI:** 10.3389/fimmu.2025.1657048

**Published:** 2025-08-20

**Authors:** Gabrielle A. Mandl, Malak Lahrichi, Perla Matar, Jamilah Abusarah, Roudy Farah, Jean Pierre Bikorimana, Abba Cisse, Moutih Rafei

**Affiliations:** ^1^ Department of Pharmacology and Physiology, Université de Montréal, Montreal, QC, Canada; ^2^ Molecular Biology Program, Université de Montréal, Montreal, QC, Canada; ^3^ Department of Microbiology, Infectious Diseases, and Immunology, Université de Montréal, Montreal, QC, Canada

**Keywords:** mesenchymal stromal cells, cancer, immunotherapy, vaccine, antigen-presenting cells

## Abstract

Mesenchymal stromal cells (MSCs) are non-hematopoietic progenitor cells that can be derived from a variety of sources including bone marrow and adipose tissues among others. MSCs are plastic adherent and easy to culture *ex vivo*, making them attractive platforms for cell-based technologies. They have an impressive immunoplasticity and can express a suppressive or inflammatory phenotype depending on their stimuli. While MSCs are mainly used in tissue regeneration or as a tool to suppress unwanted inflammation, their pro-inflammatory phenotype includes their ability to act as antigen presenting cells (APCs). This property, along with their ease of expansion and manipulation, make them excellent candidates as alternatives to dendritic cell-based technologies, especially for cancer vaccination. To generate stable MSCs with an APC-like phenotype, two main venues have been explored: genetic and pharmacological reprogramming. Routes to generating MSC-APCs have shown great promise in therapeutic and prophylactic settings *in vivo*, demonstrating effective tumor control in multiple murine models. Mechanistically, MSC-APCs appear to be generated in response to reactive oxygen species and endoplasmic reticulum stress. While much remains to be uncovered with respect to their phenotype, these reprogrammed cells show great promise as the next generation of cancer vaccine platforms. Herein, we describe the state-of-the-art in routes to reprogramming MSCs and discuss their future in the immune-oncology space as potent cancer vaccines.

## 1 Introduction

Antigen presenting cells (APCs) are essential components of innate and adaptive immunity, enabling essential processing and recognition of foreign material that must be eradicated. APCs are highly relevant for cell-based vaccine technology, as they can induce efficient, targeted, and tailored immune responses (1). In the context of cancer vaccination, APCs are usually pulsed with tumor-specific or tumor associated antigens (TSAs or TAAs, respectively) such that upon antigen processing and presentation on their cell surface, systemic administration of the vaccine would act prophylactically and/or therapeutically. This approach would not only enable a powerful measure, but has the potential to be a highly effective treatment for metastatic cancers with a major reduction in side effects (2). The optimal cellular anti-cancer vaccine would be able to overcome three major pitfalls of current vaccine technologies: 1) allowing persistent cytotoxic T lymphocyte (CTL) response without breaking immune tolerance, 2) enabling efficient antigen cross presentation, and 3) potentially promoting phagocyte-mediated efferocytosis of cancer cells (3, 4). In the context of cancer vaccination, finding the right TSA leading to the most specific and effective response remains as one of the major challenges regarding the importance of the CTL response for antitumor immunity (5, 6). Beyond that, the development of cell-based vaccine technologies that can be mass produced and used at low doses are the primary hurdle (7).

Dendritic cells (DCs) remain the most promising professional APC for generating a cell-based vaccine, as B cells and macrophages exhibit reduced T-cell activation, are more difficult to isolate and expand *ex vivo*, and have the potential to elicit deleterious inflammatory responses (8). Notably, Sipuleucel T (marketed as Provenge^®^) is the only DC-based immunotherapy that has been used to treat prostate cancer since its initial FDA approval in 2010 (9). While it has not yet shown the hoped-for therapeutic potency, this treatment illustrates the potential that APC-based technologies could have for cancer patients (10). DC therapies have also been investigated in phase III clinical trials for primary and recurrent glioblastoma and have had promising outcomes (11). Unfortunately, the development and widespread implementation of DC-based technologies has had very limited success owing to a myriad of difficulties. DCs are known to provide variable responses in different patients and environments, and are prohibitively expensive to manufacture at large scales due to difficulties with their *in vitro* isolation, manipulation, and expansion (8, 12). DCs also express PD-L1 on their surface, which can mediate T-cell inhibition, a major issue in cancer immunotherapy, as the desired prolonged immune response is needed to prevent immune evasion by cancer cells, which leads to tumor progression (13). For these reasons, there is a need to investigate alternative modalities to generating novel APC-based vaccine technologies.

## 2 Mesenchymal stromal cells: history and advancements

**Figure 1:**
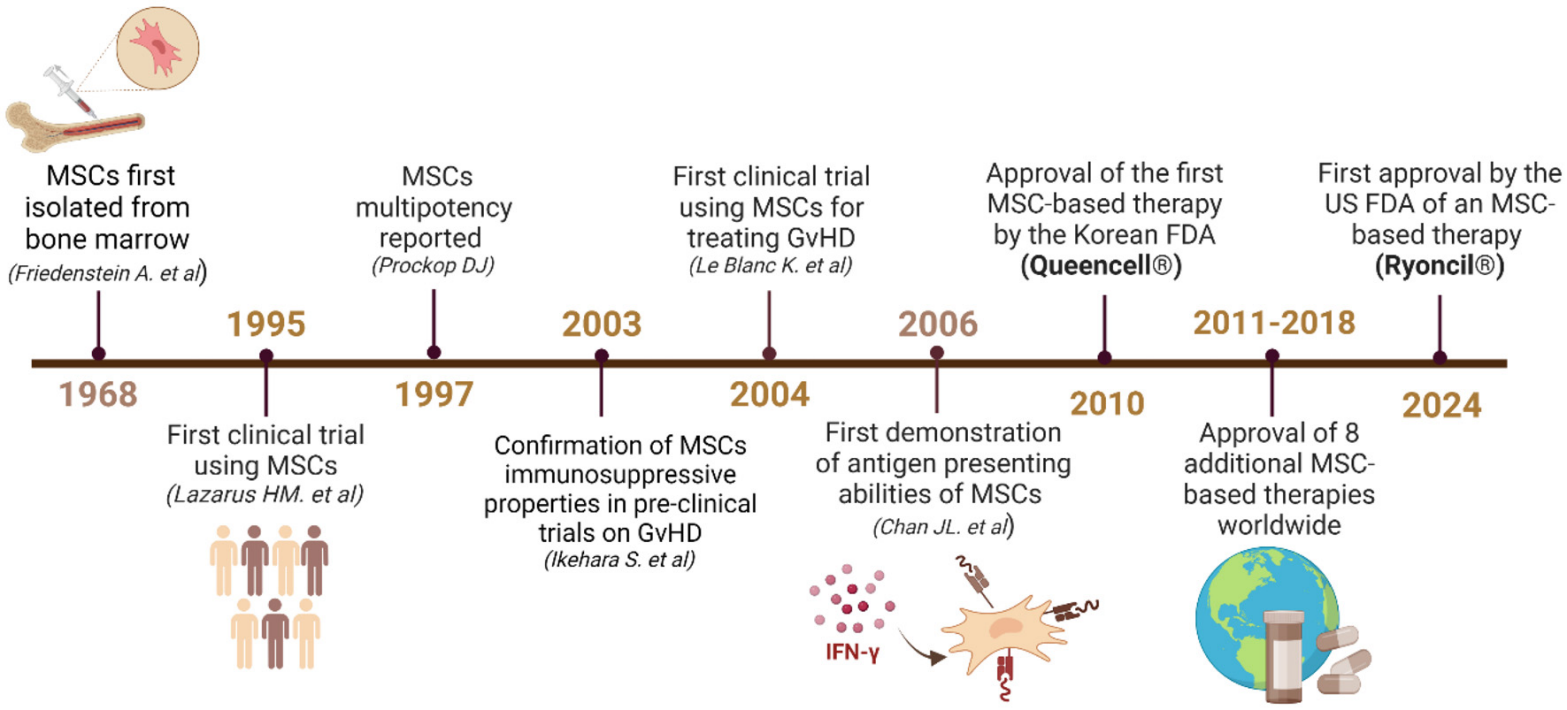
Timeline reflecting the major discoveries and advancements related to MSCs. Upon their isolation from the BM in 1968, MSCs became the center of several studies that revealed their immunosuppressive properties in 1995 and multipotency in 1997 (14–16). These discoveries enabled major advancements in regenerative therapies, which led to the first clinical trial of MSCs for GvHD (17, 18). In 2006, the antigen presenting properties of MSCs were first observed, giving rise to the advancements described in detail in this review (19). After the 2010 approval of the first MSC-based drug in South Korea (Queencell^®^), many other drugs were approved for a variety of diseases (20). In December 2024, the USFDA approved the first MSC-based therapy Ryoncil^®^ to treat acute-GvHD (21). Created in BioRender. Farah, R (22) https://BioRender.com/g36f426.

MSCs have emerged as a promising cellular technology in a variety of fields (**Figure 1**) (23–25). Initially characterized as supportive cells within the bone marrow (BM) microenvironment (26), these nonhematopoietic cells have now been identified in a variety of other tissues, and can be isolated from sources including BM, adipose tissue, and menstrual blood, among others (26, 27). Although MSCs are capable of differentiation into cells of mesenchymal origin and are able to self-renew, the term mesenchymal stromal cells is preferred to mesenchymal stem cells (28, 29). Defining criteria of MSCs include their adherence to plastic, ability to form fibroblast colonies, a spindle-like morphology, as well as their ability to differentiate into adipocytes, chondrocytes and osteoblasts (27). Their phenotypic heterogeneity has made these cells historically difficult to define, however, the International Society for Cellular therapy (ISCT) uses the aforementioned characteristics, along with a minimum phenotypic profile: expression of CD44, CD73, CD90 and CD105, while lacking expression of CD11b, CD14, CD34, CD45, CD19, CD79a, and HLA-DR (30).

Since their discovery by Friedenstein in the late 1960’s – early 1970s (14, 26), MSCs have since garnered attention for their capacity to modulate immune responses, promote tissue repair, and exert regenerative effects across various organ systems (31, 32). The majority of research on MSCs focuses on their immunosuppressive functionality, as human MSCs lack expression of CD80 and CD86, have low HLA expression, and lack expression of HLA-DR in their native state (33). MSCs have been featured in over one thousand clinical trials (clinicaltrials.gov) to date, and are perhaps best known for their important role in curing Graft-versus-Host Disease (GvHD) as first reported clinically by LeBlanc in 2004 (18). The MSC market has since expanded to include treatments for a variety of diseases, underscoring their versatility (20, 34). These products include Cartistem^®^ for the treatment of osteoarthritis in knees, Cupistem^®^ and Alofisel^®^ for Crohn’s fistula, Neuronata-R^®^ for amyotrophic lateral sclerosis, and Cellgram-AMI^®^ for acute myocardial infarction (20). Notably, Japan approved Stemirac^®^, an MSC-based therapy for spinal cord injury using auto serum-expanded autologous MSCs (35). Despite the controversial approval of the treatment (36, 37), Stemirac^®^ has been available clinically since 2019 and has sparked multiple studies on MSCs for spinal cord injury treatment (35). During the COVID-19 pandemic, MSCs also rose to prominence for treating acute respiratory distress syndrome, the main fatal complication in COVID-19 infected patients (38). Most recently, in December 2024, the United States Food and Drug Administration (USFDA) announced their first approval of an MSC-based drug, Ryocil^®^, for the treatment of acute GvHD (21).

In 2006, studies on MSCs demonstrated novel properties of these cells upon their priming with interferon-gamma (IFNγ) (39, 40). This pro-inflammatory cytokine is crucial for a variety of different immunomodulatory cascades, but is perhaps best known for playing a key role in activating macrophages and promoting T-cell differentiation (41). In the mid-2000s, several reports demonstrated that MSCs treated with IFNγ exhibit functional plasticity within a narrow window of stimulation (39, 40, 42, 43). Specifically, MSCs were found to exhibit an acquired antigen presentation capability, resulting in antigen-specific CD4 and CD8 T-cell activation and antibody production in murine models (43). Importantly, this was found to occur in a manner similar to professional APCs, such as DCs. These results sparked interest in MSCs as a potential non-hematopoietic APC candidate, as MSCs are abundant in adults, can be isolated from a variety of different tissue sources, and are extremely easy to culture *in vitro.*

Unfortunately, the reported antigen presentation properties by IFNγ-primed MSCs (MSCγ) were overshadowed by significant drawbacks. First, antigen presentation only occurred within a narrow concentration and time window of IFNγ priming, beyond which MSCs revert to their immunosuppressive behavior (19). Second, the antigen-presenting phenotype of MSCγ also exhibited concomitant expression of PD-L1, which impairs the effector functions of CTLs (43, 44). These were considered significant hurdles for the use of MSCγ as a cellular vaccine due to the possibility of generating an immunosuppressive effect when encountering an inflammatory environment following their injection *in vivo* (19, 39, 44, 45). As such, multiple investigations were carried out toward generating MSCs with the APC phenotype using alternative strategies. Herein, we aim to review the state-of-the-art in strategies for generating MSCs with antigen presentation capabilities through genetic and/or pharmacological stimulation.

## 3 Genetic reprogramming of MSCs: targeting different forms of proteasomal complexes

**Figure 2:**
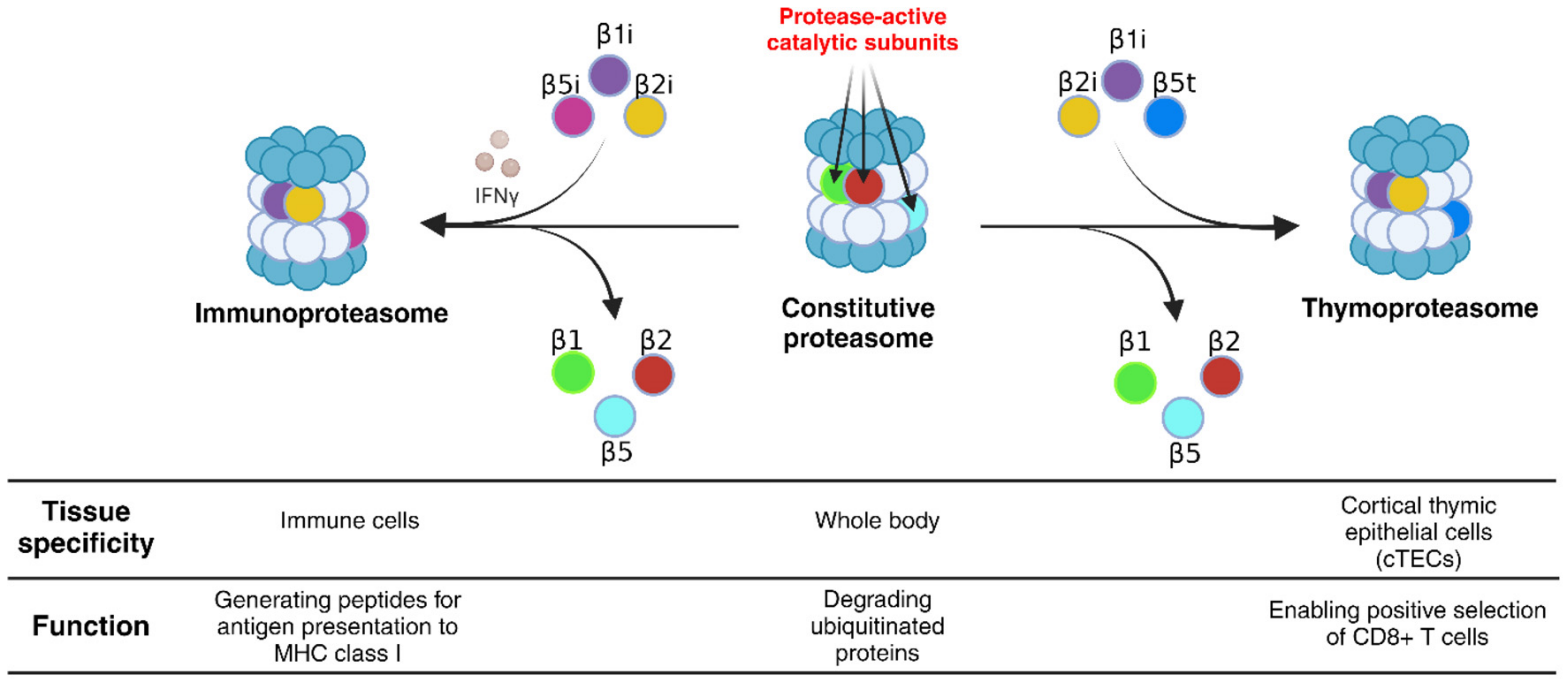
Proteasome subtypes and their tissue specificity and function. There are numerous proteasome subtypes that differ in their subunit composition, among them: the CP, the IPr and the TPr. The CP is composed of the catalytic subunits β1, β2 and β5 and is found in all cell types, since it is involved in maintaining cellular proteostasis. The IPr, with the immuno-subunits β1i, β2i and β5i, is mainly involved in immune functions such as antigen processing for presentation by APCs. The TPr, specific to the cortical thymic epithelial cells, shares the same β1i and β2i subunits of the IPr but differs by its catalytic subunit β5t, a homologue of β5 and β5i (47). Created in BioRender. Farah, R (22) https://BioRender.com/q31l729.

To overcome the shortcomings of MSCγ as a candidate for cellular vaccine development, Abusarah et al. introduced a novel approach to generating cross-presenting MSCs by modulating their proteasomal machinery (46). Proteasomes are complex macromolecular structures, for which there are three main types: 1) the constitutive proteasome (CP), which is present in all eukaryotic cells, 2) the immunoproteasome (IPr), which is found in immune cells or in response to IFNγ stimulation, and 3) the thymoproteasome (TPr), which is exclusively expressed in the cortical thymic epithelial cells (**Figure 2**) (48). These proteasomal subtypes are important for maintaining proteostasis, and for generating peptides presented by MHC-I molecules of the immune system, which are then recognized by CD8 T-cells (49, 50). Specifically, antigen degradation during an active immune response relies mainly on the IPr, while the intrathymic development of CD8 T-cells requires peptide generation by the TPr (51, 52).

**Figure 3:**
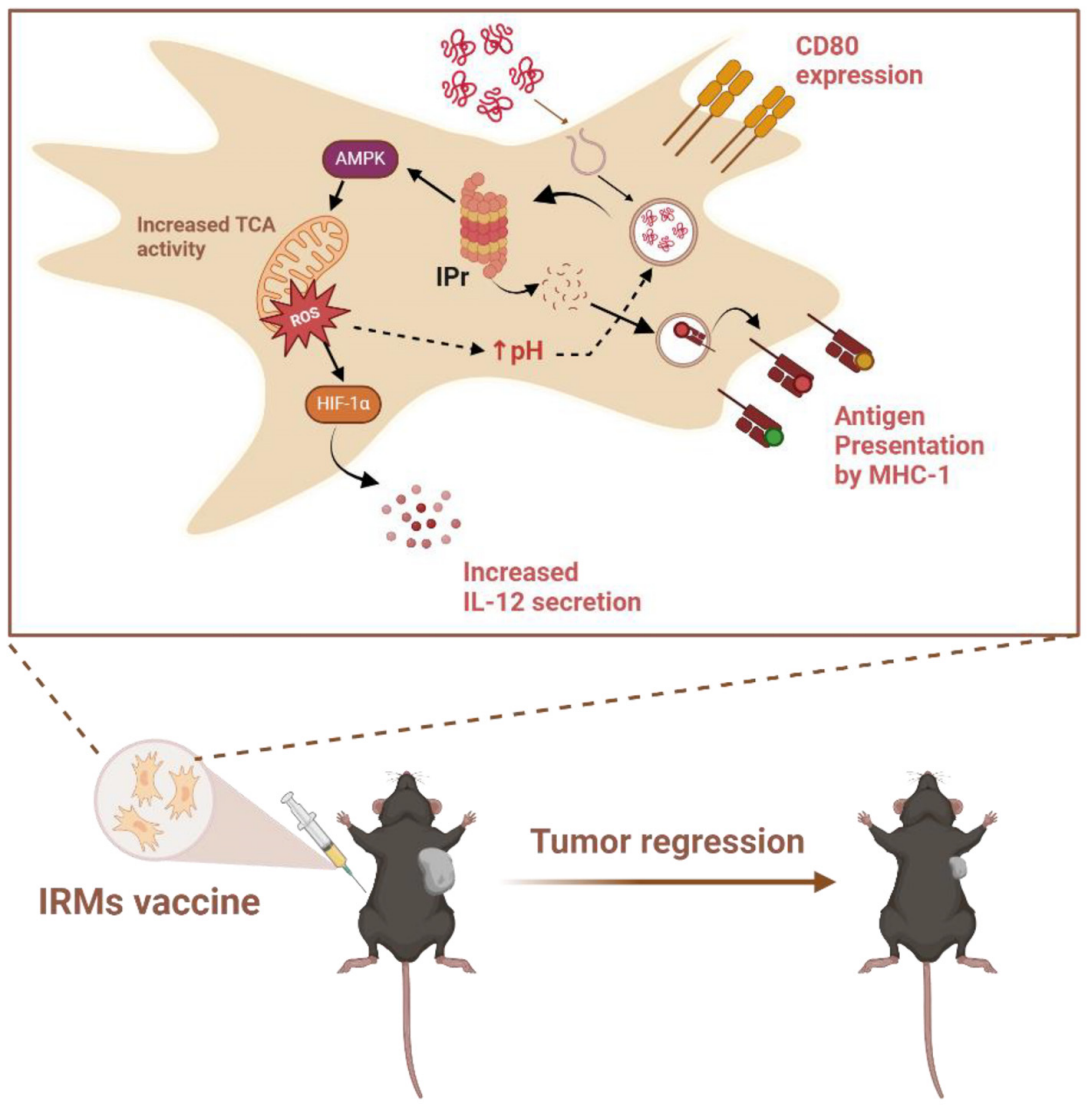
Process of antigen presentation by IRMs upon vaccination of mice. MSCs genetically modified to express the IPr present an antigen cross-presenting phenotype characterized by increased MHC-I and CD80 expression, as well as enhanced IL-12 production and secretion due to activation of the Hypoxia-inducible factor 1-alpha (HIF-1α). Improved antigen processing by the IPr was also observed, related to an increase in mitochondrial metabolic rates that helped limit intra-endosomal acidification, and thus preserve the antigen during its processing. *In vivo* administration of IRMs in C57BL/6 mice led to a strong anti-tumoral response. Created in BioRender. Farah, R (22) https://BioRender.com/r26n578.

Introduction of the IPr complex in MSCs was chosen as an initial strategy owing to its functional role in generating immunogenic peptide-MHC complexes in DCs (52). When BM-derived murine MSCs were gene-engineered to stably express the IPr complex, a plethora of changes led to APC-like properties without the need for IFNγ stimulation (46). To distinguish them from MSCγ, IPr-reprogrammed MSCs were henceforth referred to as IRMs (**Figure 3**).

The continuous expression of the IPr in MSCs led to effects beyond its reported role in antigen processing (50, 53–55), where it also caused cell-wide functional reprogramming. While IRMs maintained the basic characteristics of BM-derived MSCs, they showcased high levels of MHCI molecules (H2-K^b^, H2-D^b^ and Qa2), were positive for CD80, and negative for PD-L1 (46). The lack of PD-L1 expression is in strong contrast to MSCγ, addressing one of the major hurdles mentioned previously. Moreover, the chemokine/cytokine and gene expression profile of IRMs is closer to DCs than regular MSCs, underscoring their similarity to professional APCs. A transcriptomic analysis of IRMs was compared to that of BM-derived DCs in order to assess the potential similarities and differences that enable strong antigen cross presentation capabilities in IRMs. Indeed, IRMs were found to share similar transcriptional regulators to DCs, indicating the antigen presenting phenotype of these MSCs does mimic that of professional APCs (56). As such, IRMs were found to not only exhibit impressive antigen presenting properties, they also exhibited several capabilities which surpassed those of BM-derived DCs, making them a valid candidate for cellular vaccine development. Specifically, the differences in antigen routing and processing between IRMs and DCs translated to a more varied, wider, and 4-fold higher, repertoire of peptides presented by MHCI molecules on the surface of IRMs in comparison with DCs, potentially leading to the activation of CTLs against a distinct and wider range of tumor-derived antigens (46).

Interestingly, the antigen presentation properties of IRMs are mechanistically different from standard BM-derived DCs. Antigen uptake by IRMs is highly dependent on micropinocytosis and follows a route involving early recycling endosomes. Treatment with inhibitors of micropinocytosis abolished antigen uptake and presentation, while chloroquine, the inhibitor of early endosome acidification (57), substantially enhanced antigen cross-presentation (46). Treatment with chloroquine resulted in prolonged or delayed antigen processing, preserving the antigens from rapid degradation and leading to enhanced T-cell activation, which confirms the role of early endosomes. This is consistent with IRM’s gene expression analysis, which revealed an overall downregulation of various V-type protein ATPases of the pH reduction pathway leading to generally higher pH in the endosome of IRMs in comparison with DCs (46). This observation provides additional insight on the substantial difference in the vaccination outcome between IRMs and DCs, as it ensures limited losses of captured antigens normally induced by the high intra-endosomal acidity and protease activity (57, 58).

Although our studies provided a good comparison between IRMs and Mo-DCs (**Table 1**), investigations conducted by other groups focusing on cDC1 indicated that this subset of DCs remain the best type of APC for stimulating immunity. Future comparative studies on cDC1 biology and manufacturing are, therefore warranted to fully understand and situate the therapeutic potential of IRMs in this APC hierarchy.

**Table 1:** Comparison between IRM and DC immune potency.

	MSCs-IPr (IRMs)	Mo-DCs	cDC1s
Antigen cross-presentation	++	+	+++
Antigen uptake	+++	+++	+++
*In vivo* Survival	+++	+	+
Migration Potential	++	+	+
*In vivo* potency	+++	+	+++
Manufacturing	+++	++	+
Antigen Dosing	++	+++	+++

IRMs showed an overall strong immune activation ability as well as a good *in vivo* potency compared to Mo-DCs. On the other hand, cDC1s are better antigen cross-presenting cells but are difficult to manufacture for therapeutic interventions. +, low; ++, Medium; +++, High.

Further evaluation of the metabolic activity of IRMs reveals additional influence of IPr expression over the metabolic signature of the cells (46). In particular, the up-regulation of various metabolic genes, revealed by mRNA analysis, resulted in enhanced oxidative phosphorylation, increased oxygen consumption, and increased mitochondrial activity. This metabolic behavior was found to be central to the cross-presentation function of IRMs, as well as their enhanced T-cell activation capabilities relative to DCs. Although these metabolic changes can result in direct epigenetic modulations, it is presently unclear how exactly these changes in metabolism lead to the IRM phenotype (59). After confirming their tolerability and safety *in vivo*, IRMs were evaluated as a prophylactic vaccine *in vivo* in different murine cancer models using ovalbumin (OVA)-expressing lymphoma cells (EG.7), EL4 lymphoma or B16 melanoma cells (**Figure 3**) (46). Studies were conducted in the presence or absence of another malignant challenge and using the specific OVA antigen versus whole tumor lysate. In all the above-mentioned tests, prophylactic vaccination using IRMs demonstrated comparable or, at some points, superior anti-tumoral responses than DC vaccines. When evaluated as a therapeutic vaccine, IRM vaccination alone only delayed tumor growth, however, the co-administration of immune-checkpoint blockers and agonist stimulators provided valuable synergy with therapeutic vaccination, leading to control of established tumors and better infiltration of T-cells in the tumor (**Figure 3**) (46).

Further studies on the IRM vaccine revealed that the generated anti-tumoral immunity is reliant on both CD4 and CD8 T-cell activation and recruitment due to the loss of efficacy with the use of the inhibitor of T-cell egress from secondary lymphoid organs, FTY720 (46). However, the mechanism of action is not efferocytosis-dependent, nor is it linked to the reported migration abilities of MSCs (60), which indicates a dependency on their stable antigen cross-presenting ability, their cross priming of resident DCs and their improved *in vivo* survival post-injection to achieve the desired outcomes. Finally, in an alignment with the reported adjuvant effects of using allogenic DC vaccines (61, 62), administration of the IRM vaccine under an allogenic setting further improved the anti-tumoral response (46).

Notably, while the IPr complex is restricted to involvement in peptide generation via MHC-I molecules, MHC-II presentation was also observed upon transcriptomic analysis suggesting that IRMs may be able to mount a humoral immune response as well (46). This ability was further investigated and confirmed by Bikorimana et al. in 2022 (63). Indeed, an RNA-seq, carried out on IRMs and native MSCs, showed that 51 genes related to MHC-II antigen processing and presentation were found to be altered, such as genes related to kinesin motor proteins or the RAB-interacting lysosomal protein (*Rilp)*, further underscoring the genetic differences upon conversion from native MSCs to IRMs (63). On the other hand, sustained OVA-specific antibody titers were observed up to 8 weeks post-vaccination in Balb/c mice immunized with allogeneic OVA-pulsed C57BL/6 derived IRMs using intraperitoneal injection. Interestingly, the efficiency noted was dose and route dependent, which is in line with what has been observed for other vaccine technologies (42, 64). Furthermore, administration of IRMs with granulocyte macrophage colony stimulating factor (GM-CSF) and interleukin IL-21 cytokines simultaneously yielded a synergistic effect sustained for over 14 weeks, indicative of a potent and long-lasting response (46).

In summary, modulation of the IPr represents the first alternative strategy to IFNγ priming for generating the antigen presenting phenotype of MSCs (46). The efficacy of the IRM-based vaccine can be described as multifactorial, involving a combination of efficient antigen cross-presentation, enhanced metabolic activity, pro-inflammatory cytokine production as well as improved survival following *in vivo* delivery. Combined, these results highlight a great potential for applications in the field of personalized autologous cancer vaccines. However, altering other variants of the proteasome may also endow MSCs with greater antigen presenting capacities.

**Figure 4:**
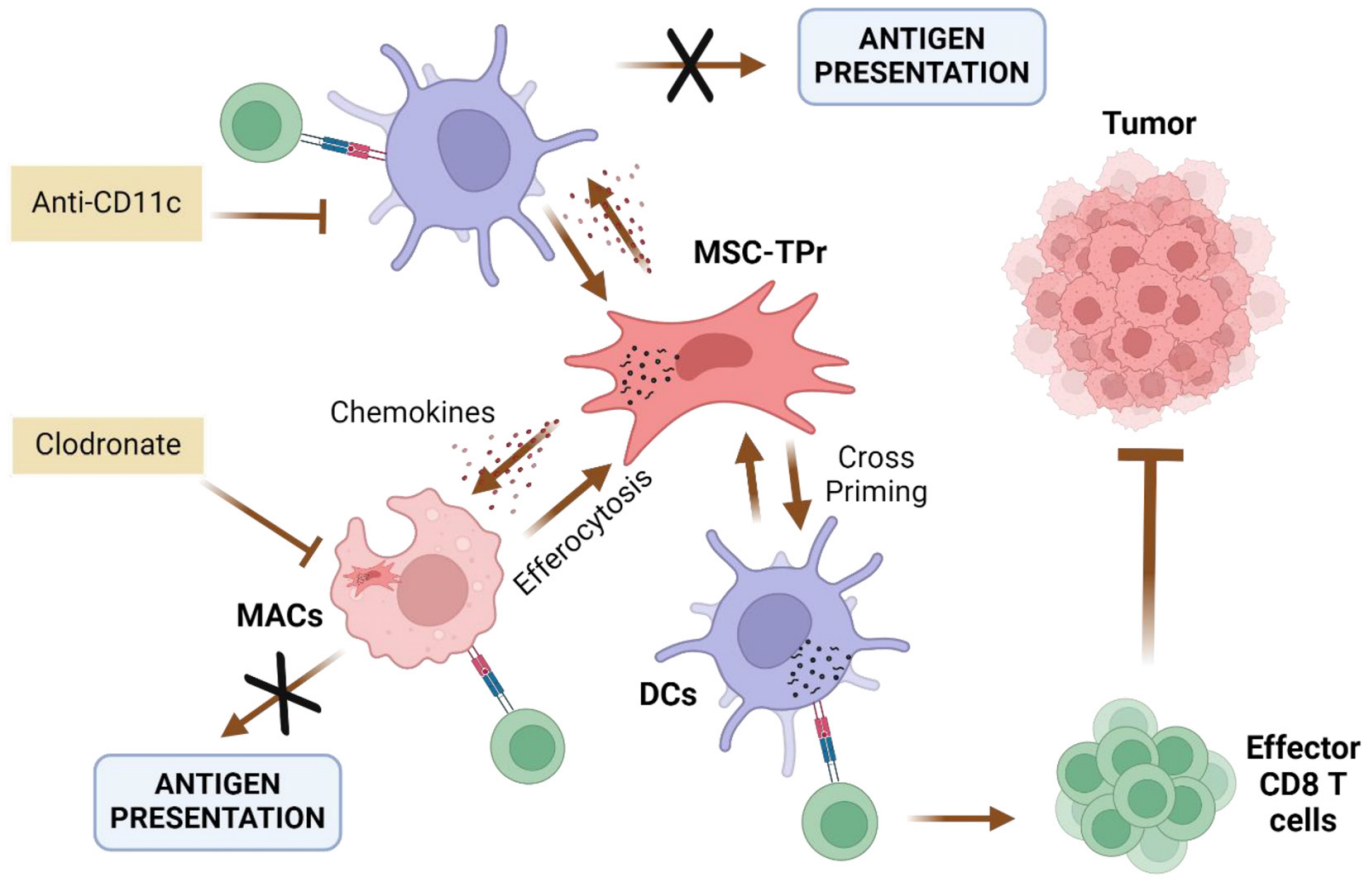
Suggested model of action for TPr-reprogrammed MSCs (MSC-TPr). Administration of MSC-TPr in immunocompetent mice induces the recruitment of Macrophages (MACs) and DCs through the secretions of soluble mediators (chemokines). The administration of anti-CD11c antibodies abrogates T-cell activation while the depletion of macrophages by clodronate leads to complete protection in challenged mice (67). Created in BioRender. Farah, R (22) https://BioRender.com/h23z032.

Many studies have demonstrated that the IPr and TPr play important roles in triggering and regulating immune responses, and several suggest that the TPr is attributed to the regulation of pro-inflammatory cytokine production (65, 66). TPr are essential for generating unique peptides associated with MHC-I molecules and developing CD8^+^ T-cells (51). Although the TPr complex is important for the positive selection of CD8 thymocytes, its contribution to peripheral T-cell immunity remains ill-defined. To this end, *Bikorimana* et al. explored whether the use of MSCs engineered to induce altered proteasomal activity through *de novo* expression of the TPr subunits could be exploited to efficiently trigger effective CD8 T-cell activation and act as a novel cancer vaccine (**Figure 4**) (67). MSCs engineered to express the TPr (MSC-TPr) were tested both *in vitro* and *in vivo* (67). Upon vaccination of a murine model, these cells demonstrated the ability to prime BM-derived DCs allowing the activation of CD8 T-cells. However, the MSCs-TPr failed to directly activate CD8 T-cells, despite transcriptional changes related to functions such as endogenous antigen presentation.

In order to understand the MSCs-TPr mechanism of action, their efferocytosis (phagocytosis by myeloid cells) was assessed. Indeed, recent discoveries showed the importance of efferocytosis for MSCs immunomodulatory effect. In fact, efferocytosis can be either triggered by apoptosis of MSCs-TPr, or through enhanced surface expression of phosphatidylserine, which serves as an “eat-me” signal for endogenous phagocytes (Macrophages, DCs) (67–69). These observations indicated that MSC-TPr play a bystander role in vaccinated animals that is reinforced by myeloid cells, in contrast to the direct role of MSC-IPr in tumor control (63, 67, 70).

Given the lack of direct T-cell activation by the TPr-MSCs, further research was devoted to improving the efficacy of IRMs. With respect to cell-based vaccines, the number of cells needed per vaccine dose represents an important consideration toward reliable and cost-effective manufacturing and distribution. For example, Provenge^®^, the only FDA-approved DC drug, requires a minimum of 50 million cells per vaccine dose, and two to three doses must be administered in a complete treatment regimen (71). Thus, a strategy was developed to decrease the number of cells needed per vaccine by trying to fine-tune the properties of IRMs (56).

**Figure 5:**
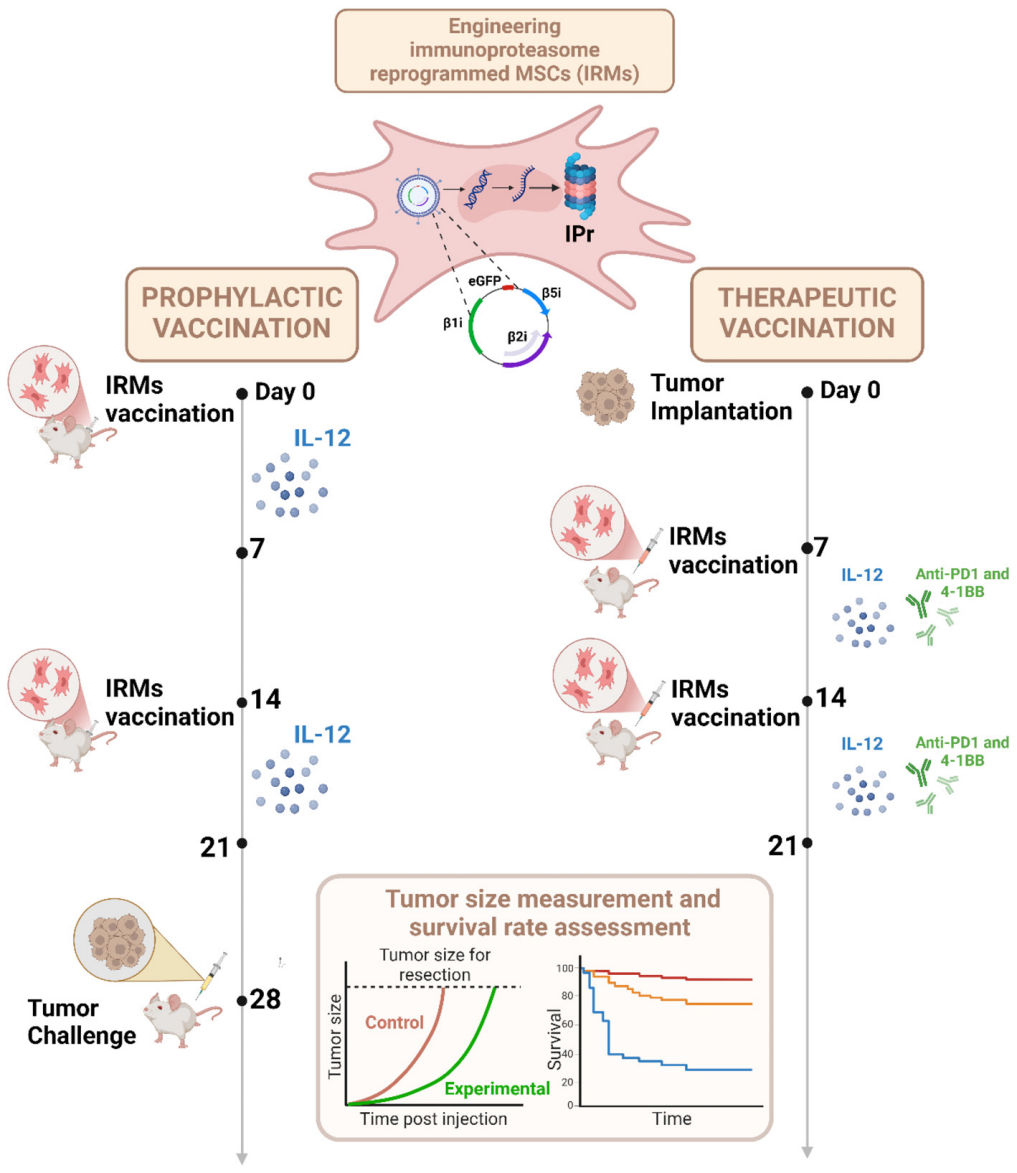
Strategies used to optimize the therapeutic potency of IRMs. MSCs were transduced by a retroviral construct composed of three inducible subunits of the murine IPr (β1i, β2i, and β5i) and the enhanced green fluorescence protein (eGFP). For *in vivo* studies, IRMs were injected prior (prophylactic vaccination) or following (therapeutic vaccination) tumor implantation. In the prophylactic design, mice were stimulated with IL-12 the week following the first and the second vaccination, before tumor challenge. For the therapeutic approach, tumor implantation was followed by two vaccinations at day seven and at day fourteen post-tumor implantation. The mice received two IL-12 and immune checkpoint inhibitors (anti-PD1 and anti-41BB) regimens after the first and second vaccination. The size of the tumors was measured during the study before the assessment of survival rate for the different vaccination strategies. Created in BioRender. Farah, R (22) https://BioRender.com/n64w077.

To achieve this, a technique called the *CIt* protocol was introduced (56). A panel of different pro-inflammatory cytokines was investigated and IL-12 was found to induce the highest cytotoxic T-cell response. Co-administration of the IRM vaccine with an IL-12 regimen was evaluated as a prophylactic vaccine strategy and found to yield a 4/10 complete response and 100% survival rate versus a 30% overall survival with IRMs alone (**Figure 5**). When tested under therapeutic settings, IRMs and IL-12 were co-administered with an anti-PD1/anti-4-1BB antibody regimen, and a 9/10 complete response was obtained (**Figure 5**). These data are highly suggestive that the combination with adjuvant immunotherapy regimens can substantially increase the efficiency of the vaccine regime. In addition, decreasing the number of cells per vaccine to only 5–000 cells/dose is remarkably low relative to DC vaccines and thus relevant for industrial scale up and practical clinical use.

**Figure 6:**
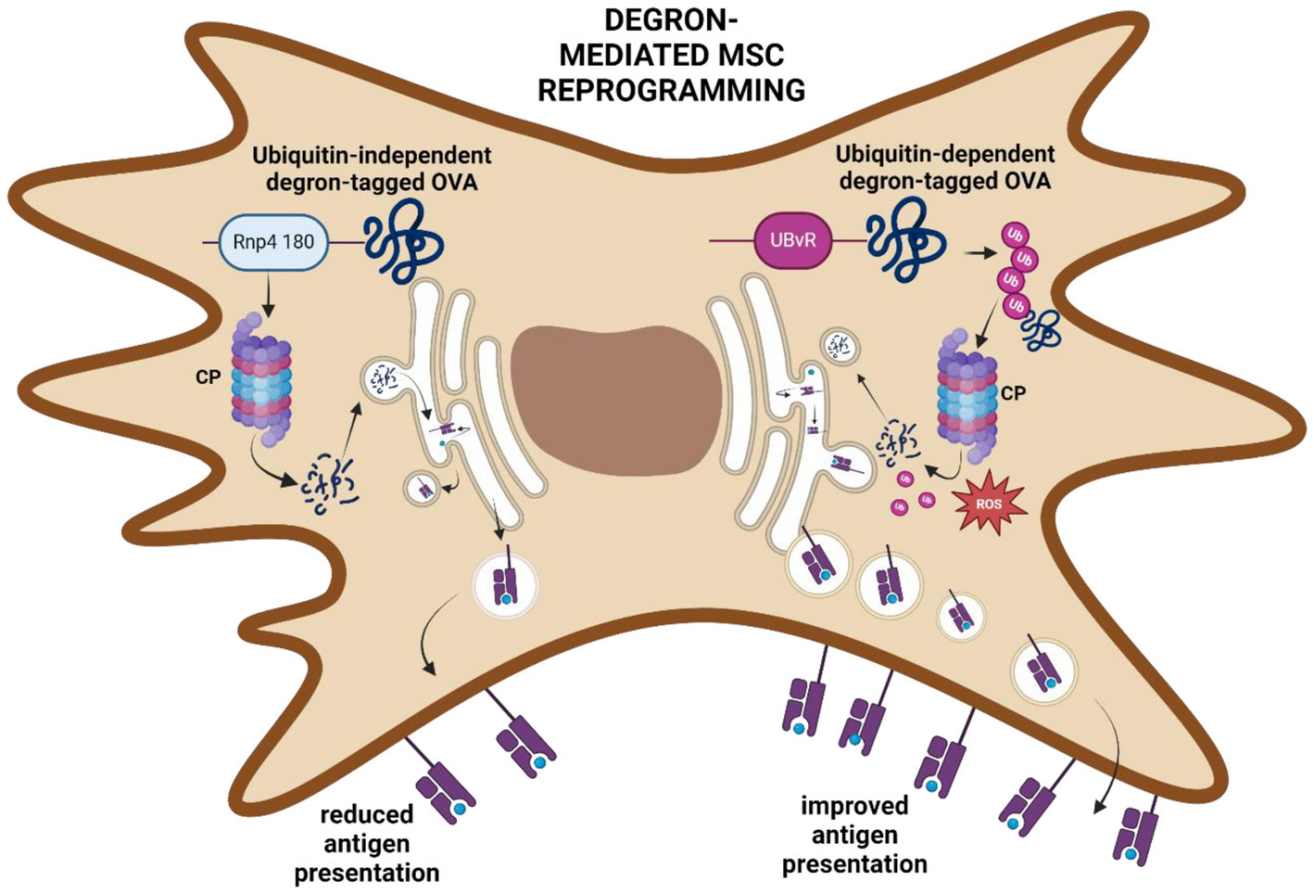
Degron-mediated reprogramming of MSCs. Rnp4 180-tagged OVA is targeted to the CP through a ubiquitin-independent mechanism leading to antigen processing and presentation. In contrast, the UBvR-tagged OVA protein is targeted to the CP through a ubiquitin-dependent pathway. UBvR-mediated processing and presentation of the OVA-derived peptides are superior to the ubiquitin-independent degron route. Created in BioRender. Farah, R (22) https://BioRender.com/t15e722.

Prior to processing by the IPr or TPr, antigens are captured by endosomes and degraded into smaller fragments (72). However, the highly acidic nature of the endosomal compartments is known to cause excessive antigen degradation, leading to loss of some important immunogenic sequences within the target antigen that could be presented to CD8 T cells if these sequences were otherwise successfully processed and presented (73, 74). Additionally, endogenously produced antigens may be prematurely released into the extracellular space upon trafficking from the ER/Golgi complex (75). Degrons represent a new tool for modulating protein content in cells (76), and were envisioned for use as a means to improve the targeting of antigens toward the CP (**Figure 6**) (77). To improve the antigen presentation properties of MSCs, two degron sequences (UBvR and Rnp4 1-80) were used to engineer MSCs to express destabilized forms of the OVA protein. Notably, the MSC-UBvR-OVA group was found to exhibit a significantly higher proportion of OVA-derived peptides at the cell surface compared to the Rnp4 1–80 degron (77). Interestingly, the major difference between UBvR and Rnp4 1–80 is the role of ubiquitination; they are ubiquitin-dependent and independent, respectively (78, 79). As such, ubiquitin was viewed as a sort of “magnet” for the degron sequences, enabling improved proteasomal processing. Mitochondrial ROS levels were also found to be significantly higher in MSC-UBvR-OVA, indicative of a direct link between ROS and degron-mediated antigen degradation and processing. This may be linked to enhanced lipid peroxidation and endosomal degradation, as observed in DCs (74). This finding adds further evidence to the notion that ROS plays a vital role in the conversion of MSCs into potent and stable APCs, as observed by the metabolic changes in IRMs (46, 56). Moreover, it suggests that the use of ubiquitin-mediated strategies to improved antigen processing, and/or introduction of a pre-destabilized antigen may represent an effective route toward tailored APCs for cancer or other diseases. Importantly, the choice of pre-destabilized antigen should be tumor-specific to limit deleterious off-target effects. As more actionable, immunogenic TSAs are discovered, techniques such as this one may rise to prominence.

Engineering MSCs to express or target different forms of proteasomal complexes enabled the production of MSC-APCs with powerful immunological properties (67, 77). The specificity with which different components of the proteasome can be altered *via* retroviral vector technology enabled a detailed understanding of how each component (IPr, TPr, and degrons for the CP) contributes to the generation of the APC phenotype. However, scaling this technology for widespread clinical use presents some logistical difficulties at the manufacturing level. Specifically, generating GMP-grade retroviral vectors, inserting them into MSCs followed by their expansion and subsequent generation of fully characterized master cell banks would be time consuming and prohibitively expensive with the current manufacturing technologies available. As such, there became a need to explore methods of generating APC-MSCs with a stable APC phenotype that could be easily scaled, highly reproducible, and cultured up to high passage numbers. Moreover, it is essential that translation from murine to human MSCs can be reliably achieved, which remains of course, a challenge in vaccine engineering strategies (80, 81). For these reasons, pharmacological reprogramming represents a straightforward strategy that should be cheaper and more amenable to murine-human translation than genetic reprogramming.

## 4 Pharmacological reprogramming of MSCs into APCs

Following the discovery of IFNγ stimulation to induce the antigen presenting phenotype of MSCs, it became attractive to investigate alternative routes to reprogramming these cells. Pharmacological reprogramming is an attractive strategy, as it is relatively simple and represents an opportunity to both achieve the desired MSC phenotype while shedding light on the pathways that must be affected in order to generate such an outcome. When using drugs to induce changes in the MSC phenotype, it is important to ensure the dose required to achieve the APC phenotype is well-tolerated by the cells and does not hinder proliferation. Moreover, pharmacological stimulation should generate a stable APC phenotype that will persist upon *in vivo* administration and should not stimulate the expression of markers like PD-L1, which are known to inhibit T-cell responses.

### 4.1 UM171a: a pyrimido-indole derivative targeting crucial genes required for antigen presentation in MSCs

The first example of MSC pharmacological reprogramming by means other than IFNγ was demonstrated in 2022 using UM171a, a drug that was known to promote the long-term expansion of hematopoetic stem cells *in vitro* (82). UM171a can stimulate an increase in the expression of genes that are crucial for antigen presentation (human leukocyte antigens A and B (HLA-A and HLA-B, H2-K/H2-D, beta 2-microglobulin (β2M), and CD86) (83). As such, it was posited that this drug may be useful for converting MSCs into APCs. Notably, UM171a demonstrated a remarkable capacity for enabling antigen cross-presentation and antigen presentation in MSCs, as observed by pulsing with OVA and the OVA-derived SIINFEKL peptide, respectively (82). In contrast to the effects of UM171a on hematopoetic stem cells, expression of the co-stimulatory molecule CD86 was not observed (82). However, this did not appear to impair antigen presentation and cross-presentation abilities, and, importantly, UM171a did not induce expression of PD-L1 on the cell surface, nor secretion of indoleamine 2,3-dioxygenase (IDO)-1 (82). This is a major advantage over IFNγ treatment, as PD-L1 and IDO-1 both act to inhibit T-cell activation, thus generating an immunosuppressive environment.

The antigen presentation effect of UM171a-treated MSCs was found to depend significantly on the levels of intracellular ROS, as pre-treatment with antioxidants MitoTEMPO and α-tocopherol disrupted antigen cross-presentation of UM171a-treated MSCs (82). Further studies demonstrated a decrease in transcription of *Psmb8* following antioxidant treatment. As *Psmb8* is responsible for expression of a major component of the IPr, the results are indicative of an essential role of this proteasomal complex in generating the desired phenotype; this is in agreement with the results obtained by Abusarah et al. by genetically reprogramming MSCs to express the IPr, as described earlier (46). Interestingly, antigen presentation was not inhibited in the presence of the antioxidants, which is indicative of an alternative pathway leading to upregulation of MHC-I following UM171a treatment that is separate from the mechanisms governing cross-presentation. As a proof-of-concept, UM171a-treated MSCs were evaluated *in vivo* in a preclinical murine T-cell lymphoma model and demonstrated an effective anti-tumoral response under a therapeutic vaccine framework. Additional studies indicated an important role in the electron transport chain (ETC)-III complex for achieving antigen cross-presentation, further underscoring the seemingly-crucial role played by ROS toward achieving the APC phenotype. Combined, the study demonstrates that UM171a treatment enabled the conversion of MSCs into the APC phenotype in a ROS-dependent fashion (82). Such ROS generation was found to induce upregulation of the IPr, indicating a central role of this machinery in generating the antigen presenting abilities of MSCs.

### 4.2 Inducing the APC phenotype by targeted inhibition of LSD1

Following the success of UM171a in generating APC-like MSCs, the parent compound of UM171a was found to target the degradation of histone H3K4, specifically *via* lysine specific demethylase 1 (LSD1) (84). LSD1 plays a critical role in demethylating lysine 4 at histone H3, and its inhibition leads to an increase in double-stranded RNA (ds-RNA) associated with upregulated expression of type I interferons and thus a pro-inflammatory state (84). Inhibition of LSD1 is known for enhancing anti-tumoral T-cell responses, and promoting tumor immunogenicity. As such, Mardani et al. postulated that inhibition of LSD1 *via* administration of tranylcypromine hydrochloride (TC), a potent monoamine oxidase inhibitor and irreversible inhibitor of LSD1, may also enable conversion of MSCs to the APC phenotype (85).

Upon TC-treatment of MSCs, a marked increase in the cell surface expression of H2-K^b^ was observed at levels similar to UM171a-treated cells (82, 85). Akin to UM171a-treated MSCs, TC-treated cells also did not secrete IDO-1 (85). However, in contrast to UM171a-treated cells, expression of the endothelial protein C receptor was not increased, and ROS levels were significantly lower in TC-treated cells, suggesting UM171a and TC exert their effects via different modes of action. In depth analysis further demonstrated upregulation of MHC-I, as well as IFNα and IFNβ signaling ((*Ifi6*, *Ifi35*, *Ifit1*, *Oas3*, *Irf1*, *Irf9*, *Bst2*, *Jak1*, *Isg15*, *Ifnar2*, *Ifnar1*, and *Ptpn1*) (85). Importantly, increased expression of the IPr subunits β1i (*Psmb9*), β2i (*Psmb10*) and β5i (*Psmb8*) were observed, while the expression level of the CP (*Psmb5, Psmb6*, and *Psmb7*) was not altered (85). These results further corroborate the important role played by the IPr in generating antigen presentation properties in MSCs.

In contrast to UM171a, TC treatment was found to enable antigen presentation as observed by SIINFEKL pulsing and subsequent B3Z activation, but did not induce antigen cross-presentation capabilities in MSCs upon pulsing with OVA (85). The effective antigen presentation was consistent with the increased MHC-I levels, and subsequent investigations demonstrated TC treatment enhances antigen presentation capabilities of MSCs by stabilizing cell surface peptide:MHC complexes. Following these results, anti-cancer vaccination protocols were developed using an EG.7 T-cell lymphoma model. A prophylactic approach was explored, whereby naïve C57BL/6 mice (H2^b^) were subcutaneously injected with a low dose of syngeneic MSCs at 0 and 14 days. One-week post-immunization (day 21), the mice were challenged with EG.7 T-cell lymphoma. A powerful effect was observed for the TC-treated, SIINFEKL-pulsed MSC group, yielding an 80% survival rate. Both groups receiving MSCs treated with TC alone or MSC pulsing with SIINFEKL alone yielded comparable results of delayed tumor growth and 20% survival rate in comparison with the control groups (16 vs 25 days). Despite some delay in tumor growth, this outcome leaves both test groups behind in comparison with TC-treated, SIINFEKL-pulsed MSC group (85). Of importance, however, the delay in tumor growth for the TC-treated group (without SIINFEKL) suggests that IFNβ secretion from TC-treated MSCs may prime a local endogenous immune response without the need for pre-treating the MSCs with antigen pulsing. Central memory (T_CM_) and effector (T_eff_) CD4 and CD8 T-cell populations were also quantified for all groups, with a significant increase in the percentage of CD8 T_eff_ cells observed in the TC-treated and TC+SIINFEKL-treated groups, corroborating these hypotheses (85).

LSD1 inhibition via TC treatment provides additional confirmation of multiple routes to pharmacological reprogramming of MSCs into APCs. Of interest, the ability of TC treatment to enable antigen presentation, but not cross presentation, may serve as a foundation for in-depth exploration of which components of the APC-like phenotype can be induced, depending upon the properties of the pharmacological compound being used.

### 4.3 Targeting endosomal escape to elicit an APC phenotype

Following the discovery that IRMs relied on early endosomal recycling (46), and that ROS played a crucial role on UM171a-mediated APC functions in MSCs (82), Goncalves et al. postulated these should be primary targets for pharmacological reprogramming (86). While not completely understood at this time, the accumulated knowledge of inducing transformation of MSCs into APCs has clearly suggested endosomal recycling and ROS production to play a central role. For optimal use of APC-like MSCs as anti-cancer vaccines, antigen presentation and cross-presentation are both desired. Given the role of endosomes in antigen uptake and degradation, it was postulated that a pharmacologically active compound, which could promote early endosomal escape may enable efficient conversion of MSCs into APCs.

Accum^®^ is a recently-introduced technology designed to promote enhanced intracellular drug delivery by promoting endosomal escape of cargo into the cytosol (87–89). Under this framework, it had been previously demonstrated that Accum^®^ conjugated to OVA enabled potent antigen presentation in DCs, enabling a strong antitumoral effect (90). As such, the technology represented an interesting route to pharmacological reprogramming of MSCs. Accum^®^ is composed of a nuclear localization sequence and bile acid moiety, each of which can be modified to generate different Accum^®^ variants with different properties. One of the generated variants, known as A1, was demonstrated to successfully convert MSCs into APCs upon admixing with OVA (86). Notably, this is a convenient result, as there was no need to chemically conjugate the antigen to the A1 molecule. In theory, this means the A1 variant should enable successful conversion to the APC phenotype in the presence of virtually any antigen, and is highly scalable, since a specific functional group does not need to be present to chemically conjugate the two components.

A1 treatment led to reprogramming of over 1500 genes, underscoring the powerful impact elicited by the technology (86). Of major importance, several genes related to the unfolded protein response (UPR) were upregulated, suggesting an important role of ER stress in modulating the APC phenotype of A1-reprogrammed MSCs (henceforth referred to as ARMs) (86). Mechanistically, A1 was confirmed to trigger endosomal escape as evidenced by pulsing A1-treated MSCs with exogenous cytochrome C, which can only induce apoptosis as an intact antigen (91). Studies on the role of ROS production in eliciting the APC phenotype confirmed endosomal ROS production and subsequent lipid peroxidation are critical to achieving antigen cross-presentation (86). Interestingly, intra-endosomal ROS, but not mitochondrial-derived ROS, were found to be crucial for inducing the APC phenotype, in contrast to what was observed for UM171a-treated MSCs (82, 86). This difference suggests that there are multiple sources for ROS production that can generate APC-like MSCs; additional studies are required to further understand this aspect. Moreover, given the role of ROS in inducing protein damage (and thus activating the UPR), a powerful interplay between these facets of the physiology of ARMs is apparent.

To illustrate the potency of this anti-cancer vaccine, the ARM cells were pulsed with tumor lysate rather than a single defined antigen (86). This approach enables multiple advantages: i) targeting a single antigen is inevitably less specific and may promotes tumor adaptation/escape leading to treatment tolerance, and ii) there is no known single TSA shared by a large set of individuals at this time. As such, using tumor lysate not only enables multiple antigens to be presented, but it also allows a patient-specific mix of antigens to be processed and expressed on the cell surface (92). Given the high degree of antigen variability within a single tumor, this adaptable approach represents an interesting solution to personalized medicine. Subcutaneous administration of ARM cells to mice implanted with solid lymphoma tumors was performed as a monotherapy and in combination with anti-PD-1. The adjuvant anti-PD-1/ARM treatment yielded a powerful tumor control response, with 80% of mice surviving past the 40-day mark (86). Notably, ARM administration alone improved outcomes relative to anti-PD-1 treatment, with significant delays in tumor progression and 40% overall survival by day 40. Importantly, the study was performed using both syngeneic and allogeneic ARM cells, with the allogeneic vaccine found to be more effective. The latter point is encouraging given the fact that it may enable large-scale vaccine production in a time-efficient manner (86). Despite these improvements, the remaining hurdle toward implementation of the ARM vaccine remained the relatively high dose of antigen administration to MSCs required to achieve CD8 T cell activation (0.5 mg/mL). As such, further research efforts were aimed at discovering analogues of A1 that elicited the same powerful effects, but at lower antigen doses.

**Figure 7:**
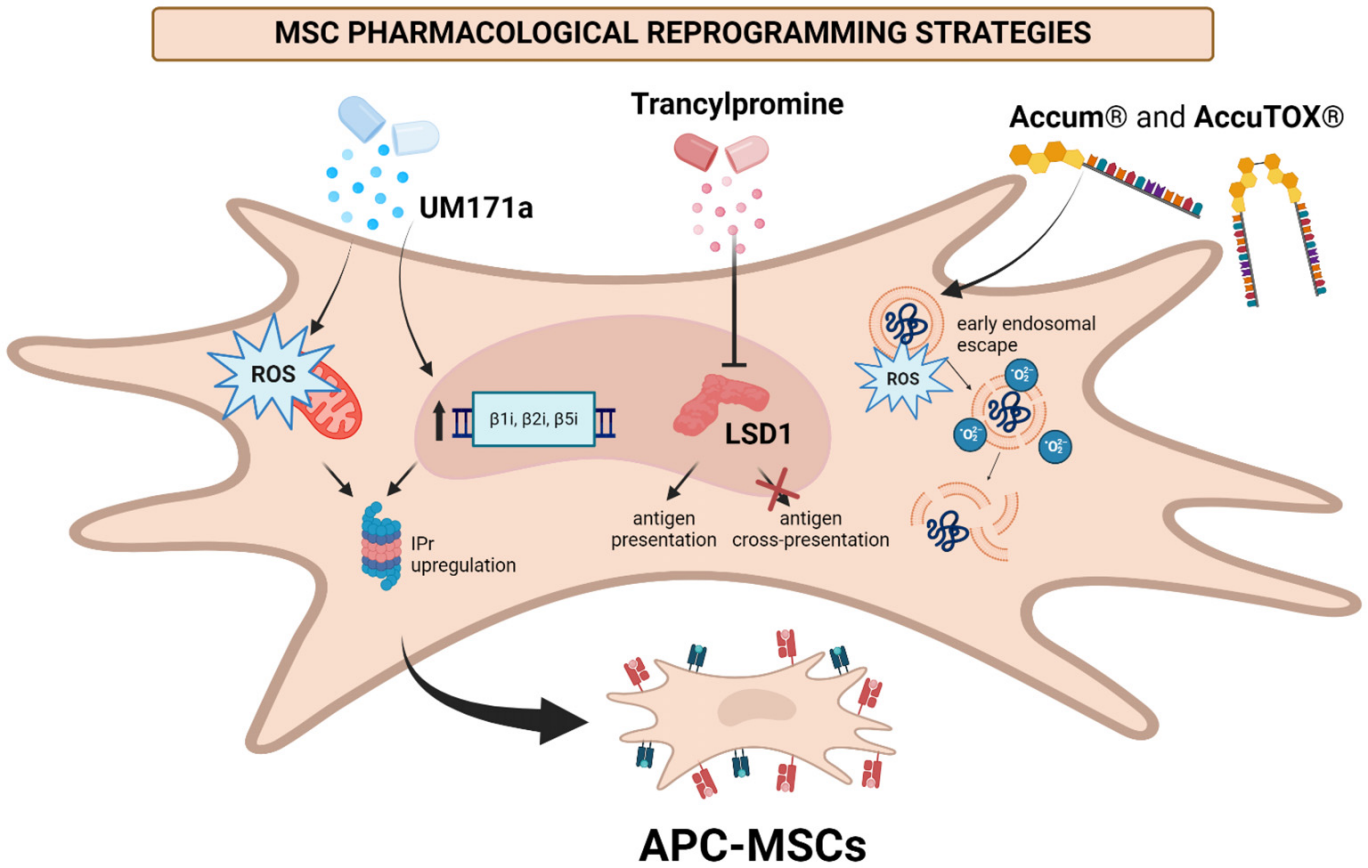
Summary of pharmacological reprogramming modalities used to convert MSCs into APCs. UM171a induces mitochondrial ROS production, which seems to support the antigen presenting phenotype of MSCs. Inhibition of LSD1 following TC administration converts MSCs into APCs by stabilizing the peptide-MHCI complexes at the cell surface. A1 and AccuTOX^®^ promote early endosome escape via ROS production as well as lipid peroxidation, and activates the UPR to convert MSCs into APCs (82, 85, 86). Created in BioRender. Farah, R (22) https://BioRender.com/m78f755.

Recently, the same team introduced AccuTOX^®^, a dimeric analogue of A1 (**Figure 7**). Impressively, AccuTOX^®^ maintained the desired characteristics of early endosomal escape and ROS production, but required a 10-fold smaller dose of antigen (0.05 mg/mL) to achieve MSC conversion to the APC phenotype relative to the dose needed when using A1 (86, 93). This was demonstrated in both murine and human MSCs, an important consideration for clinical translation. The reduction in antigen dose was attributed to an improvement in antigen uptake, processing, and presentation, as well as activation of the IRE-1α/XPB-1 pathway involved in the UPR. Interestingly, unlike A1, the extracellular formation of protein aggregates was not required to elicit antigen presentation, suggesting that UPR activation may be a main driver in the MSC-APC conversion process, and implicates the XPB-1/IRE-1α pathway as a driver of antigen cross-presentation capabilities. Along these lines, active processing of XBP-1 was observed in CD8^+^ cDC1 cells in the absence of ER stress (58). The second generation MSC-based vaccine generated using the AccuTOX^®^ derivative, called ARM-X, was tested on murine models of melanoma (B16F0), pancreatic (Pan02 cells) and colon (CT26 cells) cancer, and no side effects beyond mild inflammation at the injection sites were observed. The ARM-X vaccine was administered in combination with anti-PD-1 antibodies, where 80%, 90% and 60% survival was obtained for the melanoma, pancreatic and colon cancer models, respectively (93). Moreover, it was observed that *in vivo* T-cell activation was strongly affected by antigen pulsing dose. As such, future studies should consider the role of antigen dose when developing MSC-based vaccines.

Combined, these results underscore the potency of MSCs as APCs and the powerful potential of pharmacological reprogramming toward yielding an anticancer vaccine modality (**Figure 7**). The implications of A1 and AccuTOX^®^ treatment on ROS production, endosomal escape and UPR induction suggest a central role of these components toward generating an APC-like phenotype in culture-adapted MSCs. Until the exact mechanisms governing antigen presentation/cross-presentation in MSCs are fully understood, future studies should aim to draw on the pharmacological properties of the drugs used to obtain these promising results in order to home in on the most powerful routes for inducing antigen presentation and cross-presentation properties in MSCs.

## 5 Conclusions and outlook

Despite a promising *in vitro* anti-tumoral response, the currently-tested DC-cell based vaccines have presented limited clinical efficacy (8, 12). In addition to their transient antigen presenting abilities, DC vaccines exhibit many hurdles owing to the need for large numbers of cells per vaccine dose due to the small percentage of DC that successfully reach the patients lymph nodes, and the possible immune tolerance DCs can induce in the tumor microenvironment (94). Because of these challenges, clinical studies on new DC vaccine approaches have led to inconclusive results in phase III trials, despite promising results in phases I and II (94).

To address these limitations, the use of MSCs is being explored as a new alternative strategy for cell-based vaccines. Known for their immunomodulatory abilities, MSCs were shown to be conditional APCs under a specific IFNγ treatment regimen (19, 43). However, a switch of the MSCs’ antigen presenting characteristics into immunosuppressive proprieties was noted upon exposure to higher or sustained IFNγ doses (19). This major issue drove the need for different approaches to generating MSCs with a stable APC phenotype. Of the many strategies available, genetically engineering MSCs to express the IPr complex was initially explored and was further enhanced by a combination of immune checkpoints inhibitors and cytokines (*CIt* protocol), leading to the need for only 5–000 cells per dose (46, 56). The use of ubiquitin-dependent degron sequences was also shown to successfully induce an APC-like MSC phenotype, acting as a strategy to target the CP that exists in all cell types (77). Interestingly, reprogramming MSCs via TPr expression did not directly induce CD8 T-cell activation, suggesting very specific roles for the different subsets of proteasomes in eliciting an APC phenotype in MSCs (67). While the genetic engineering strategies were largely successful, cell engineering remains challenging, especially in human MSCs and scales relevant for manufacturing. Although pharmacological reprogramming is less sophisticated than gene engineering, it represents an elegant approach because of its inherent simplicity. Notably, the first successful pharmacological reprogramming study reported the use of UM171A, a hematopoietic agent, capable of inducing an increase in MSCs antigen cross-presentation capabilities (82). Encouraging results were also obtained using A1 and TC, which have distinct mechanisms of action from one another, and from UM171a (82, 85, 86). However, each of the studies are in agreement, in that they all implicate ROS production and ER stress in the conversion of MSCs into antigen presenting cells (46, 63, 77, 86). The observed activation of the IRE-1α/XPB-1 axis of the UPR following A1 or AccuTOX^®^ administration further corroborates these findings, and suggests we are becoming closer to understanding the mechanistic aspects of MSC conversion to the APC phenotype (93). These hypotheses are corroborated by studies in the literature related to the antigen presentation capacity of DCs (95–98). Specifically, the role of the UPR in driving cDC1 functions has become a prominent new finding (58). With regard to MSCs, the next step would be to explore the link between UPR and antigen presentation/cross-presentation in MSCs through pharmacological stimulation. By understanding the mechanisms that trigger a robust APC-MSC response it will become possible to study a library of drugs which act on these pathways and may successfully stimulate an APC-like MSC phenotype. In turn, this will enable the potential for customized approaches to rapidly generate cell-based anticancer vaccines for multiple indications.

The secretome of these APC-like MSCs likely plays a major role in their efficacy, as the secretion of inhibitory cytokines and chemokines could have a deleterious effect on T-cell activation. While extensive studies of the APC-MSC secretome have not been carried out owing to the nascency of the field, preliminary findings show a pro-inflammatory cytokine/chemokine profile following MSC reprogramming using the strategies discussed above. Notably, IRMs produce significantly higher amounts of IL-12 than DCs, and also exhibit *de novo* production of chemokine (C-X-C motif) ligand 1 (CXCL1) and lipopolysaccharide-induced CXC chemokine (LIX) (48). Compared to DCs, however, IL-4 and IL-10 production was not observed. Similarly, UM171a-treated MSCs show significant increases in M-CSF, GM-CSF, IL-6, IP10, KC, LIX, and MIP-2, and also exhibit lower levels of IDO relative to IFNγ-treated MSCs (82). Combined, this suggests APC-MSCs exhibit a pro-inflammatory secretome regardless of the mode of reprogramming. Further studies should be carried out to determine the universality of this conclusion as well as how different modes of reporgramming can affect the MSC-APC secretome. MSCs are also known for secreting high amounts of extracellular vesicles (EVs), which are rapidly being recognized for their potential in new vaccine technologies (99). Interestingly, at this time, there are no reports studying EVs from APC-MSCs, to our knowledge. Given the well-known role of EVs secreted from professional APCs toward modulating immune responses, there is almost certainly a rich body of knowledge to be discovered surrounding EVs in the APC-MSC space (99). Moreover, the use of EVs instead of whole cells can be considerably more cost-effective, and as such, APC-MSC-derived EVs may be of benefit when considering large-scale manufacturing of these products.

Lastly, the interaction of APC-MSCs with the broader immune landscape such as natural killer (NK) cells, Treg cells, myeloid-derived suppressor cells, and macrophages, among others, also constitutes a major consideration. To date, in-depth studies on APC-MSC interactions with these different immune players is lacking, to our knowledge. As mentioned before, efferocytosis by endogenous phagocytes appears to play a central role in the efficacy of some APC-MSCs (67, 77, 86, 93) but not others (48), underscoring the importance of studying these interactions such that a clearer understanding can be gained. Notably, MSC-TPr rely on efferocytosis from CD11^hi^ phagocytes and cross-priming with DCs to elicit their therapeutic activity (67). We encourage those in the field to begin investigating these interactions in more detail, as it is almost certain that understanding APC-MSCs within the broader immune context will provide new directions toward amplifying APC-MSC efficacy.

The versatile nature of MSCs has brought these cells to the forefront of cancer immunotherapy in the past two decades. Their unique capacity to act as both immunostimulatory and immunosuppressive renders them highly attractive for treating a myriad of diseases. The field has only just begun to develop approaches to reliably generate MSCs with APC capacities, but the opportunities brought forward are exciting and suggest a bright future for MSCs in addressing the issues brought forth with DCs in cancer immunotherapy. As more fundamental understanding is gained on the biochemistry governing the immunological properties of these cells, we are confident the field will see a renaissance in cell-based therapies with numerous opportunities for improving treatment outcomes of patients worldwide.

## References

[1] Liu J Fu M Wang M Wan D Wei Y Wei X. Cancer vaccines as promising immuno-therapeutics: platforms and current progress. J Hematol Oncol. (2022) 15:1–26. doi: 10.1186/s13045-022-01247-x, PMID: 35303904 PMC8931585

[2] Luo W Yang G Luo W Cao Z Liu Y Qiu J. Novel therapeutic strategies and perspectives for metastatic pancreatic cancer: Vaccine therapy is more than just a theory. Cancer Cell Int. (2020) 20:1–10. doi: 10.1186/s12935-020-1147-9, PMID: 32158356 PMC7057654

[3] Mocellin S Mandruzzato S Bronte V Lise M Nitti D. Part I: Vaccines for solid tumours. Lancet Oncol. (2004) 5:681–9. doi: 10.1016/S1470-2045(04)01610-9, PMID: 15522656

[4] Rosenberg SA Yang JC Restifo NP. Cancer immunotherapy: Moving beyond current vaccines. Nat Med. (2004) 10:909–15. Available online at: https://www.nature.com/articles/nm1100 (Accessed January 15, 2025). 10.1038/nm1100 PMC1435696 15340416

[5] Peri A Salomon N Wolf Y Kreiter S Diken M Samuels Y. The landscape of T cell antigens for cancer immunotherapy. Nat Cancer. (2023) 4:937–54. Available online at: https://www.nature.com/articles/s43018-023-00588-x (Accessed January 15, 2025). 10.1038/s43018-023-00588-x 37415076

[6] Malacopol AT Holst PJ. Cancer vaccines: recent insights and future directions. Int J Mol Sci. (2024) 25:11256. Available online at: https://www.mdpi.com/1422-0067/25/20/11256/htm (Accessed January 15, 2025)., PMID: 39457036 10.3390/ijms252011256 PMC11508577

[7] Thomas F. Overcoming vaccine development challenges. Pharm Technol. (2021) 45:24–8.

[8] Anguille S Smits EL Lion E Van Tendeloo VF Berneman ZN. Clinical use of dendritic cells for cancer therapy. Lancet Oncol. (2014) 15:257–67. doi: 10.1016/S1470-2045(13)70585-0 24872109

[9] Kantoff PW Higano CS Shore N Berger E Small EJ. Sipuleucel T immunotherapy for CRPC. New Engl J. (2010) 363:441–22.

[10] Huber ML Haynes L Parker C Iversen P. Interdisciplinary Critique of Sipuleucel-T as immunotherapy in castration-resistant prostate cancer. J Natl Cancer Inst. (2012) 104:273–9. doi: 10.1093/jnci/djr514, PMID: 22232132 PMC3283534

[11] Liau LM Ashkan K Brem S Campian JL Trusheim JE Iwamoto FM. Association of Autologous Tumor Lysate-Loaded Dendritic Cell Vaccination with Extension of Survival among Patients with Newly Diagnosed and Recurrent Glioblastoma: A Phase 3 Prospective Externally Controlled Cohort Trial. JAMA Oncol. (2023) 9:112–21. Available online at: https://jamanetwork.com/journals/jamaoncology/fullarticle/2798847 (Accessed January 15, 2025)., PMID: 36394838 10.1001/jamaoncol.2022.5370 PMC9673026

[12] Fu C Ma T Zhou L Mi QS Jiang A. Dendritic cell-based vaccines against cancer: challenges, advances and future opportunities. Immunol Investigat. (2022) 51:2133–58. doi: 10.1080/08820139.2022.2109486, PMID: 35946383

[13] Wang Q Qin Y Li B. CD8+ T cell exhaustion and cancer immunotherapy. Cancer Lett. (2023) 559:216043. doi: 10.1016/j.canlet.2022.216043, PMID: 36584935

[14] Friedenstein AJ Jakovlevich A Petrakova K Kurolesova A Frolova G. Heterotopic transplants of bone marrow. Transplantation. (1968) 6:230–47. doi: 10.1097/00007890-196803000-00009 5654088

[15] Prockop DJ. Marrow stromal cells as stem cells for nonhematopoietic tissues. Science (80-). (1997) 276:71–4. doi: 10.1126/science.276.5309.71, PMID: 9082988

[16] Lazarus HM Haynesworth SE Gerson SL Rosenthal NS Caplan AI. Ex vivo expansion and subsequent infusion of human bone marrow-derived stromal progenitor cells (mesenchymal progenitor cells): implications for therapeutic use. Bone Marrow Transplant. (1995) 16(4):557–64., PMID: 8528172

[17] Ikehara S. Treatment of autoimmune diseases in mice by a new method for allogeneic bone marrow transplantation. Ther Apher Dial. (2003) 7:292–7. doi: 10.1046/j.1526-0968.2003.00060.x, PMID: 12924603

[18] Le Blanc K Rasmusson I Sundberg B Götherström C Hassan M Uzunel M. Treatment of severe acute graft-versus-host disease with third party haploidentical mesenchymal stem cells. Lancet. (2004) 363:1439–41. doi: 10.1016/S0140-6736(04)16104-7 15121408

[19] Chan JL Tang KC Patel AP Bonilla LM Pierobon N Ponzio NM. Antigen-presenting property of mesenchymal stem cells occurs during a narrow window at low levels of interferon-γ. Blood. (2006) 107:4817–24. doi: 10.1182/blood-2006-01-0057, PMID: 16493000 PMC1895812

[20] Barrera-Barrera LE Barrera-Saldaña SA Mesenchymal HA Fernández-Garza LE Barrera-Barrera SA Barrera-Saldaña HA. Mesenchymal Stem Cell Therapies Approved by Regulatory Agencies around the World. Pharm. (2023) 16:1334. Available online at: https://www.mdpi.com/1424-8247/16/9/1334/htm (Accessed January 15, 2025)., PMID: 37765141 10.3390/ph16091334 PMC10536665

[21] FDA approves remestemcel-L-rknd for steroid-refractory acute graft versus host disease in pediatric patients | FDA . Available online at: https://www.fda.gov/drugs/resources-information-approved-drugs/fda-approves-remestemcel-l-rknd-steroid-refractory-acute-graft-versus-host-disease-pediatric (Accessed January 15, 2025).

[22] Farah R. Timeline of discoveries and advancements related to mesenchymal stromal cells. Available online at: https://biorender.com/g36f426.

[23] Müller L Tunger A Wobus M von Bonin M Towers R Bornhäuser M. Immunomodulatory properties of mesenchymal stromal cells: an update. Front Cell Dev Biol. (2021) 9:1–9. doi: 10.3389/fcell.2021.637725, PMID: 33634139 PMC7900158

[24] García-Bernal D García-Arranz M Yáñez RM Hervás-Salcedo R Cortés A Fernández-García M. The current status of mesenchymal stromal cells: controversies, unresolved issues and some promising solutions to improve their therapeutic efficacy. Front Cell Dev Biol. (2021) 9:1–18. doi: 10.3389/fcell.2021.650664, PMID: 33796536 PMC8007911

[25] Wobma H Satwani P. Mesenchymal stromal cells: Getting ready for clinical primetime. Transfus Apher Sci. (2021) 60:103058. doi: 10.1016/j.transci.2021.103058, PMID: 33495081

[26] Friedenstein AJ Chailakhjan RK Lalykina KS. The development of fibroblast colonies in monolayer cultures of Guinea-pig bone marrow and spleen cells. Cell Prolif. (1970) 3:393–403. doi: 10.1111/j.1365-2184.1970.tb00347.x, PMID: 5523063

[27] Nombela-Arrieta C Ritz J Silberstein LE. The elusive nature and function of mesenchymal stem cells. Nat Rev Mol Cell Biol. (2011) 12:126–31. Available online at: https://www.nature.com/articles/nrm3049 (Accessed January 15, 2025)., PMID: 21253000 10.1038/nrm3049 PMC3346289

[28] Lindner U Kramer J Rohwedel J Schlenke P. Mesenchymal stem or stromal cells: Toward a better understanding of their biology? Transfus Med Hemother. (2010) 37:75–83. doi: 10.1159/000290897, PMID: 20737049 PMC2914415

[29] Viswanathan S Shi Y Galipeau J Krampera M Leblanc K Martin I. Mesenchymal stem versus stromal cells: International Society for Cell & Gene Therapy (ISCT^®^) Mesenchymal Stromal Cell committee position statement on nomenclature. Cytotherapy. (2019) 21:1019–24. Available online at: http://www.isct-cytotherapy.org/article/S1465324919308412/fulltext (Accessed January 15, 2025). 10.1016/j.jcyt.2019.08.002 31526643

[30] Dominici M Le Blanc K Mueller I Slaper-Cortenbach I Marini FC Krause DS. Minimal criteria for defining multipotent mesenchymal stromal cells. The International Society for Cellular Therapy position statement. Cytotherapy. (2006) 8:315–7. Available online at: http://www.isct-cytotherapy.org/article/S1465324906708817/fulltext (Accessed January 15, 2025)., PMID: 16923606 10.1080/14653240600855905

[31] Rasmusson I. Immune modulation by mesenchymal stem cells. Exp Cell Res. (2006) 312:2169–79. doi: 10.1016/j.yexcr.2006.03.019, PMID: 16631737

[32] Jiang W Xu J. Immune modulation by mesenchymal stem cells. Cell Prolif. (2020) 53:1–16. doi: 10.1111/cpr.12712, PMID: 31730279 PMC6985662

[33] Stagg J. Immune regulation by mesenchymal stem cells: Two sides to the coin. Tissue Antigens. (2007) 69:1–9. doi: 10.1111/j.1399-0039.2006.00739.x, PMID: 17212702

[34] Levy O Kuai R Siren EMJ Bhere D Milton Y Nissar N. Shattering barriers toward clinically meaningful MSC therapies. Sci Adv. (2020) 6. doi: 10.1126/sciadv.aba6884, PMID: 32832666 PMC7439491

[35] Mou C Wang X Li W Li Z Liu N Xu Y. Efficacy of mesenchymal stromal cells intraspinal transplantation for patients with different degrees of spinal cord injury: A systematic review and meta-analysis. Cytotherapy. (2023) 25:530–6. doi: 10.1016/j.jcyt.2023.01.012, PMID: 36805381

[36] Japan should put the brakes on stem-cell sales. Nature. (2019) 565:535–6. doi: 10.1038/d41586-019-00332-5, PMID: 30700883

[37] Miyamoto S. Japan responds: stem-cell therapy justified. Nature. (2019) 569:40. doi: 10.1038/d41586-019-01364-7, PMID: 31040418

[38] Mesoblast’s Stem Cell Therapy Shows 83% Survival in Ventilator-Dependent COVID-19 Patients - BioSpace . Available online at: https://www.biospace.com/mesoblast-ltd-s-stem-cell-therapy-shows-83-percent-survival-in-covid-19-patients (Accessed January 15, 2025).

[39] Caplan AI. Adult mesenchymal stem cells for tissue engineering versus regenerative medicine. J Cell Physiol. (2007) 213:341–7. doi: 10.1002/jcp.21200, PMID: 17620285

[40] Figueroa FE Carrión F Villanueva S Khoury M. Mesenchymal Stem Cell treatment for autoimmune diseases: A critical review. Biol Res. (2012) 45:269–77. Available online at: http://www.scielo.cl/scielo.php?script=sci_arttext&pid=S0716-97602012000300008&lng=en&nrm=iso&tlng=en (Accessed January 15, 2025)., PMID: 23283436 10.4067/S0716-97602012000300008

[41] Alspach E Lussier DM Schreiber RD. Interferon γ and its important roles in promoting and inhibiting spontaneous and therapeutic cancer immunity. Cold Spring Harb Perspect Biol. (2019) 11:a028480. doi: 10.1101/cshperspect.a028480, PMID: 29661791 PMC6396335

[42] Tomchuck SL Norton EB Garry RF Bunnell BA Morris CA Freytag LC. Mesenchymal stem cells as a novel vaccine platform. Front Cell Infect Microbiol. (2012) 2:140. Available online at: www.frontiersin.org (Accessed January 15, 2025)., PMID: 23162801 10.3389/fcimb.2012.00140 PMC3499769

[43] Stagg J Pommey S Eliopoulos N Galipeau J. Interferon-γ-stimulated marrow stromal cells: A new type of nonhematopoietic antigen-presenting cell. Blood. (2006) 107:2570–7. doi: 10.1182/blood-2005-07-2793, PMID: 16293599

[44] François M Romieu-Mourez R Stock-Martineau S Boivin M-N Bramson JL Galipeau J. Mesenchymal stromal cells cross-present soluble exogenous antigens as part of their antigen-presenting cell properties. Blood. (2009) 114:2632–8. doi: 10.1182/blood-2009-02-207795, PMID: 19654411

[45] Sheng H Wang Y Jin Y Zhang Q Zhang Y Wang L. A critical role of IFNγ in priming MSC-mediated suppression of T cell proliferation through up-regulation of B7-H1. Cell Res. (2008) 18:846–57. doi: 10.1038/cr.2008.80, PMID: 18607390

[46] Abusarah J Khodayarian F El-Hachem N Salame N Olivier M Balood M. Engineering immunoproteasome-expressing mesenchymal stromal cells: A potent cellular vaccine for lymphoma and melanoma in mice. Cell Rep Med. (2021) 2:100455. doi: 10.1016/j.xcrm.2021.100455, PMID: 35028603 PMC8714858

[47] Tanaka K Mizushima T Saeki Y. The proteasome: Molecular machinery and pathophysiological roles. Biol Chem. (2012) 393:217–34. doi: 10.1515/hsz-2011-0285, PMID: 23029643

[48] Watanabe A Yashiroda H Ishihara S Lo M Murata S. The molecular mechanisms governing the assembly of the immuno-and thymoproteasomes in the presence of constitutive proteasomes. Cells. (2022) 11:1580. Available online at: https://www.mdpi.com/2073-4409/11/9/1580/htm (Accessed January 15, 2025)., PMID: 35563886 10.3390/cells11091580 PMC9105311

[49] Heink S Ludwig D Kloetzel PM Krüger E. IFN-γ-induced immune adaptation of the proteasome system is an accelerated and transient response. Proc Natl Acad Sci U.S.A. (2005) 102:9241–6. doi: 10.1073/pnas.0501711102, PMID: 15944226 PMC1166598

[50] De Verteuil D Muratore-Schroeder TL Granados DP Fortier MH Hardy MP Bramoullé A. Deletion of immunoproteasome subunits imprints on the transcriptome and has a broad impact on peptides presented by major histocompatibility complex I molecules. Mol Cell Proteomics. (2010) 9:2034–47. Available online at: https://pmc.ncbi.nlm.nih.gov/articles/PMC2938112/., PMID: 20484733 10.1074/mcp.M900566-MCP200 PMC2938112

[51] Murata S Sasaki K Kishimoto T Niwa SI Hayashi H Takahama Y. Regulation of CD8+ T cell development by thymus-specific proteasomes. Science (80-). (2007) 316:1349–53. doi: 10.1126/science.1141915, PMID: 17540904

[52] Basler M Kirk CJ Groettrup M. The immunoproteasome in antigen processing and other immunological functions. Curr Opin Immunol. (2013) 25:74–80. doi: 10.1016/j.coi.2012.11.004, PMID: 23219269

[53] Toes REM Nussbaum AK Degermann S Schirle M Emmerich NPN Kraft M. Discrete cleavage motifs of constitutive and immunoproteasomes revealed by quantitative analysis of cleavage products. J Exp Med. (2001) 194:1–12. doi: 10.1084/jem.194.1.1, PMID: 11435468 PMC2193442

[54] Kerkmann-Tucek A Banat G-A Cochlovius B Zöller M. Antigen loss variants of a murine renal cell carcinoma: Implications for tumor vaccination. Int J Cancer. (1998) 77:114–22. doi: 10.1002/(SICI)1097-0215(19980703)77:1<114::AID-IJC18>3.0.CO, PMID: 9639402

[55] Parmiani G Castelli C Dalerba P Mortarini R Rivoltini L Marincola FM. Cancer immunotherapy with peptide-based vaccines: what have we achieved? Where are we going? JNCI J Natl Cancer Inst. (2002) 94:805–18. doi: 10.1093/jnci/94.11.805, PMID: 12048268

[56] Bikorimana JP El-Hachem N Abusarah J Eliopoulos N Talbot S Shammaa R. The CIt protocol: A blueprint to potentiate the immunogenicity of immunoproteasome-reprogrammed mesenchymal stromal cells. iScience. (2022) 25:105537. doi: 10.1016/j.isci.2022.105537, PMID: 36437872 PMC9682353

[57] Abdul Alim Al-Bari M Md Abdul Alim Al-Bari C. Targeting endosomal acidification by chloroquine analogs as a promising strategy for the treatment of emerging viral diseases. Pharmacol Res Perspect. (2017) 5:e00293. doi: 10.1002/prp2.293, PMID: 28596841 PMC5461643

[58] García-González P Fernández D Gutiérrez D Parra-Cordero M Osorio F. Human cDC1s display constitutive activation of the UPR sensor IRE1. Eur J Immunol. (2022) 52:1069–76. doi: 10.1002/eji.202149774, PMID: 35419836 PMC9541385

[59] Qin S Xie B Wang Q Yang R Sun J Hu C. New insights into immune cells in cancer immunotherapy: from epigenetic modification, metabolic modulation to cell communication. MedComm [Internet]. (2024) 5:e551. doi: 10.1002/mco2.551, PMID: 38783893 PMC11112485

[60] Wang C Li Z Zhu Z Chai Y Wu Y Yuan Z. Allogeneic dendritic cells induce potent antitumor immunity by activating KLRG1+CD8 T cells. Sci Rep. (2019) 9:1–14. Available online at: https://www.nature.com/articles/s41598-019-52151-3 (Accessed January 15, 2025)., PMID: 31664180 10.1038/s41598-019-52151-3 PMC6820535

[61] Fotaki G Jin C Kerzeli IK Ramachandran M Martikainen MM Karlsson-Parra A. Cancer vaccine based on a combination of an infection-enhanced adenoviral vector and pro-inflammatory allogeneic DCs leads to sustained antigen-specific immune responses in three melanoma models. Oncoimmunology. (2018) 7. doi: 10.1080/2162402X.2017.1397250, PMID: 29399398 PMC5790347

[62] Fotaki G Jin C Ramachandran M Kerzeli IK Karlsson-Parra A Yu D. Pro-inflammatory allogeneic DCs promote activation of bystander immune cells and thereby license antigen-specific T-cell responses. Oncoimmunology. (2018) 7. doi: 10.1080/2162402X.2017.1395126, PMID: 29399392 PMC5790348

[63] Bikorimana JP Abusarah J Salame N El-Hachem N Shammaa R Rafei M. Humoral immunity to allogeneic immunoproteasome-expressing mesenchymal stromal cells requires efferocytosis by endogenous phagocytes. Cells. (2022) 11:1–15. doi: 10.3390/cells11040596, PMID: 35203247 PMC8869887

[64] Zhang L Wang W Wang S. Effect of vaccine administration modality on immunogenicity and efficacy. Expert Rev Vaccines. (2015) 14:1509–23. doi: 10.1586/14760584.2015.1081067, PMID: 26313239 PMC4915566

[65] Goetzke CC Ebstein F Kallinich T. Role of proteasomes in inflammation. J Clin Med. (2021) 10:1783. Available online at: https://www.mdpi.com/2077-0383/10/8/1783/htm (Accessed January 15, 2025). 33923887 10.3390/jcm10081783 PMC8072576

[66] Murata S Takahama Y Kasahara M Tanaka K. The immunoproteasome and thymoproteasome: functions, evolution and human disease. Nat Immunol. (2018) 19:923–31. doi: 10.1038/s41590-018-0186-z, PMID: 30104634

[67] Bikorimana JP El-Hachem N El-Kadiry AEH Abusarah J Salame N Shammaa R. Thymoproteasome-expressing mesenchymal stromal cells confer protective anti-tumor immunity via cross-priming of endogenous dendritic cells. Front Immunol. (2021) 11:1–12. doi: 10.3389/fimmu.2020.596303, PMID: 33542714 PMC7853649

[68] Wang J Li J Yin L Wang X Dong Y Zhao G. MSCs promote the efferocytosis of large peritoneal macrophages to eliminate ferroptotic monocytes/macrophages in the injured endometria. Stem Cell Res Ther. (2024) 15:1–15. doi: 10.1186/s13287-024-03742-z, PMID: 38693589 PMC11064342

[69] Ghahremani Piraghaj M Soudi S Ghanbarian H Bolandi Z Namaki S Hashemi SM. Effect of efferocytosis of apoptotic mesenchymal stem cells (MSCs) on C57BL/6 peritoneal macrophages function. Life Sci. (2018) 212:203–12. doi: 10.1016/j.lfs.2018.09.052, PMID: 30287233

[70] Bikorimana JP Saad W Abusarah J Lahrichi M Talbot S Shammaa R. CD146 defines a mesenchymal stromal cell subpopulation with enhanced suppressive properties. Cells. (2022) 11:1–12. doi: 10.3390/cells11152263, PMID: 35892560 PMC9331786

[71] Experience CT. Provenge^®^ (sipuleucel-T) Prescribing Information Highlights. Seal Beach: Dendreon Pharmaceuticals, LLC (2017).

[72] Kloetzel PM. Antigen processing by the proteasome. Nat Rev Mol Cell Biol. (2001) 2:179–88. Available online at: https://www.nature.com/articles/35056572 (Accessed January 15, 2025). 10.1038/35056572 11265247

[73] Jancic C Savina A Wasmeier C Tolmachova T El-Benna J Dang PMC. Rab27a regulates phagosomal pH and NADPH oxidase recruitment to dendritic cell phagosomes. Nat Cell Biol. (2007) 9:367–78. Available online at: https://www.nature.com/articles/ncb1552 (Accessed January 15, 2025)., PMID: 17351642 10.1038/ncb1552

[74] Dingjan I Verboogen DRJ Paardekooper LM Revelo NH Sittig SP Visser LJ. Lipid peroxidation causes endosomal antigen release for cross-presentation. Sci Rep. (2016) 6. Available online at: https://www.nature.com/articles/srep22064., PMID: 26907999 10.1038/srep22064 PMC4764948

[75] Wu LG Hamid E Shin W Chiang HC. Exocytosis and endocytosis: Modes, functions, and coupling mechanisms*. Annu Rev Physiol. (2014) 76:301–31. doi: 10.1146/annurev-physiol-021113-170305, PMID: 24274740 PMC4880020

[76] Natsume T Kanemaki MT. Conditional degrons for controlling protein expression at the protein level. Annu Rev Genet. (2017) 51:83–102. doi: 10.1146/annurev-genet-120116-024656, PMID: 29178817

[77] Bikorimana JP Farah R Abusarah J Mandl GA Erregragui MA Pereira M. Forced intracellular degradation of xenoantigens as a novel modality for cell-based cancer immunotherapy(2024). Available online at: https://www.ssrn.com/abstract=5013530 (Accessed January 15, 2025)., PMID: 10.1016/j.isci.2025.111957 PMC11889607 40060894

[78] Chassin H Müller M Tigges M Scheller L Lang M Fussenegger M. A modular degron library for synthetic circuits in mammalian cells. Nat Commun. (2019) 10:1–11. Available online at: https://www.nature.com/articles/s41467-019-09974-5 (Accessed January 15, 2025)., PMID: 31043592 10.1038/s41467-019-09974-5 PMC6494899

[79] Ha SW Ju D Xie Y. The N-terminal domain of Rpn4 serves as a portable ubiquitin-independent degron and is recognized by specific 19S RP subunits. Biochem Biophys Res Commun. (2012) 419:226–31. doi: 10.1016/j.bbrc.2012.01.152, PMID: 22349505 PMC3906847

[80] Khanna R Burrows SR. Human immunology: a case for the ascent of non-furry immunology. Immunol Cell Biol. (2011) 89:330–1. doi: 10.1038/icb.2010.173, PMID: 21412238

[81] Maeng H Terabe M Berzofsky JA. Cancer vaccines: translation from mice to human clinical trials. Curr Opin Immunol. (2018) 51:111–22. doi: 10.1016/j.coi.2018.03.001, PMID: 29554495 PMC5943163

[82] Salame N Bikorimana JP El-Hachem N Saad W Kurdi M Zhao J. UM171A-induced ROS promote antigen cross-presentation of immunogenic peptides by bone marrow-derived mesenchymal stromal cells. Stem Cell Res Ther. (2022) 13:1–15. doi: 10.1186/s13287-021-02693-z, PMID: 35012668 PMC8751335

[83] Chagraoui J Lehnertz B Girard S Spinella JF Fares I Tomellini E. UM171 induces a homeostatic inflammatory-detoxification response supporting human HSC self-renewal. PloS One. (2019) 14:e0224900. doi: 10.1371/journal.pone.0224900, PMID: 31703090 PMC6839847

[84] Subramaniam A Žemaitis K Talkhoncheh MS Yudovich D Bäckström A Debnath S. Lysine-specific demethylase 1A restricts ex vivo propagation of human HSCs and is a target of UM171. Blood. (2020) 136:2151–61. doi: 10.1182/blood.2020005827, PMID: 32582923 PMC7645986

[85] Mardani F Saad W El-Hachem N Bikorimana JP Kurdi M Shammaa R. LSD1 inhibition enhances the immunogenicity of mesenchymal stromal cells by eliciting a dsRNA stress response. Cells. (2022) 11. doi: 10.3390/cells11111816, PMID: 35681511 PMC9180800

[86] Gonçalves MP Farah R Bikorimana J-P Abusarah J EL-Hachem N Saad W. A1-reprogrammed mesenchymal stromal cells prime potent antitumoral responses. iScience. (2024) 27. doi: 10.1016/j.isci.2024.109248, PMID: 38433914 PMC10907831

[87] Bikorimana JP El-Hachem N Moreau M Lawson C Tai LH Gonçalves M. Intratumoral administration of unconjugated Accum™ impairs the growth of pre-established solid lymphoma tumors. Cancer Sci. (2023) 00:1–12. doi: 10.1111/cas.15985, PMID: 37776054 PMC10728015

[88] Bikorimana JP Abusarah J Gonçalves M Farah R Saad W Talbot S. An engineered Accum-E7 protein-based vaccine with dual anti-cervical cancer activity. Cancer Sci. (2024) 115:1102–13. doi: 10.1111/cas.16096, PMID: 38287511 PMC11007051

[89] El-Kadiry AEH Beaudoin S Plouffe S Rafei M. Accum™ Technology: A novel conjugable primer for onco-immunotherapy. Mol. (2022) 27:3807. Available online at: https://www.mdpi.com/1420-3049/27/12/3807/htm (Accessed January 15, 2025)., PMID: 35744930 10.3390/molecules27123807 PMC9227040

[90] Bikorimana JP Salame N Beaudoin S Balood M Crosson T Abusarah J. Promoting antigen escape from dendritic cell endosomes potentiates anti-tumoral immunity. Cell Rep Med. (2022) 3. Available online at: http://www.cell.com/article/S2666379122000349/fulltext., PMID: 35492876 10.1016/j.xcrm.2022.100534 PMC9040180

[91] Jemmerson R LaPlante B Treeful A. Release of intact, monomeric cytochrome c from apoptotic and necrotic cells. Cell Death Differ. (2002) 9:538–48. Available online at: https://www.nature.com/articles/4400981 (Accessed January 15, 2025)., PMID: 11973612 10.1038/sj.cdd.4400981

[92] Diao L Liu M. Rethinking antigen source: cancer vaccines based on whole tumor cell/tissue lysate or whole tumor cell. Adv Sci. (2023) 10:2300121. doi: 10.1002/advs.202300121, PMID: 37254712 PMC10401146

[93] Bikorimana JP El-Hachem N Mandl GA Stanga D Abusarah J Farah R. ARM-X: An adaptable mesenchymal stromal cell-based vaccination platform suitable for solid tumors. BioRXiv. (2025). doi: 10.21203/rs.3.rs-5828115/v1, PMID: 40660347 PMC12261777

[94] Hurwitz AA Watkins SK. Immune suppression in the tumor microenvironment: A role for dendritic cell-mediated tolerization of T cells. Cancer Immunol Immunother. (2012) 61:289–93. doi: 10.1007/s00262-011-1181-5, PMID: 22237887 PMC6948839

[95] Cubillos-Ruiz JR Silberman PC Rutkowski MR Chopra S Perales-Puchalt A Song M. ER stress sensor XBP1 controls anti-tumor immunity by disrupting dendritic cell homeostasis. Cell. (2015) 161:1527–38. Available online at: http://www.cell.com/article/S0092867415005759/fulltext (Accessed January 15, 2025)., PMID: 26073941 10.1016/j.cell.2015.05.025 PMC4580135

[96] Manoury B Maisonneuve L Podsypanina K. The role of endoplasmic reticulum stress in the MHC class I antigen presentation pathway of dendritic cells. Mol Immunol. (2022) 144:44–8. doi: 10.1016/j.molimm.2022.02.007, PMID: 35184022

[97] Salvagno C Cubillos-Ruiz JR. The impact of endoplasmic reticulum stress responses in dendritic cell immunobiology. Int Rev Cell Mol Biol. (2019) 349:153–76. doi: 10.1016/bs.ircmb.2019.08.004, PMID: 31759430

[98] Kotsias F Hoffmann E Amigorena S Savina A. Reactive oxygen species production in the phagosome: Impact on antigen presentation in dendritic cells. Antioxid Redox Signal. (2013) 18:714–29. doi: 10.1089/ars.2012.4557, PMID: 22827577

[99] Robbins PD Morelli AE. Regulation of immune responses by extracellular vesicles. Nat Rev Immunol. (2014) 14:195–208. doi: 10.1038/nri3622, PMID: 24566916 PMC4350779

